# Privacy-Preserving Methods for Feature Engineering Using Blockchain: Review, Evaluation, and Proof of Concept

**DOI:** 10.2196/13600

**Published:** 2019-08-14

**Authors:** Michael Jones, Matthew Johnson, Mark Shervey, Joel T Dudley, Noah Zimmerman

**Affiliations:** 1 Center for Biomedical Blockchain Research Icahn School of Medicine at Mount Sinai Redwood City, CA United States; 2 Institute for Next Generation Healthcare Icahn School of Medicine at Mount Sinai New York City, NY United States

**Keywords:** privacy, machine learning, confidentiality, data collection, mobile health, feature engineering, geolocation, blockchain, smart contract, cryptography, trusted execution environment

## Abstract

**Background:**

The protection of private data is a key responsibility for research studies that collect identifiable information from study participants. Limiting the scope of data collection and preventing secondary use of the data are effective strategies for managing these risks. An ideal framework for data collection would incorporate feature engineering, a process where secondary features are derived from sensitive raw data in a secure environment without a trusted third party.

**Objective:**

This study aimed to compare current approaches based on how they maintain data privacy and the practicality of their implementations. These approaches include traditional approaches that rely on trusted third parties, and cryptographic, secure hardware, and blockchain-based techniques.

**Methods:**

A set of properties were defined for evaluating each approach. A qualitative comparison was presented based on these properties. The evaluation of each approach was framed with a use case of sharing geolocation data for biomedical research.

**Results:**

We found that approaches that rely on a trusted third party for preserving participant privacy do not provide sufficiently strong guarantees that sensitive data will not be exposed in modern data ecosystems. Cryptographic techniques incorporate strong privacy-preserving paradigms but are appropriate only for select use cases or are currently limited because of computational complexity. Blockchain smart contracts alone are insufficient to provide data privacy because transactional data are public. Trusted execution environments (TEEs) may have hardware vulnerabilities and lack visibility into how data are processed. Hybrid approaches combining blockchain and cryptographic techniques or blockchain and TEEs provide promising frameworks for privacy preservation. For reference, we provide a software implementation where users can privately share features of their geolocation data using the hybrid approach combining blockchain with TEEs as a supplement.

**Conclusions:**

Blockchain technology and smart contracts enable the development of new privacy-preserving feature engineering methods by obviating dependence on trusted parties and providing immutable, auditable data processing workflows. The overlap between blockchain and cryptographic techniques or blockchain and secure hardware technologies are promising fields for addressing important data privacy needs. Hybrid blockchain and TEE frameworks currently provide practical tools for implementing experimental privacy-preserving applications.

## Introduction

### Background

#### Data Privacy Issues With New Technologies

The emergence of social networks, smartphones, wearable devices, and internet of things (IoT) devices introduces unprecedented avenues for the mass collection of personal data about behaviors, biology, and health. The ubiquity of these technologies presents novel challenges when considering how to protect the privacy of individuals, and the potential to reveal sensitive and identifiable information intentionally or unintentionally has grown.

A recent Pew Research Center report found that physical location data represent one of the most sensitive data types [1]; yet more than 1000 popular smartphone apps track precise location data, some of which sell that data to third parties for targeted ads or analytics [2]. Prompts that grant an app permission to collect location data rarely reflect how the data will be used, with specifics buried in an app’s privacy policy. Although location companies claim that the data collected are used to analyze aggregate patterns, not individual identities, employees and clients still have access to raw data and could identify users without their consent. Major telecommunications carriers sell user location data, and reporters have shown that data can be resold to a long chain of downstream companies. The lack of regulation in this data ecosystem has resulted in a black market for the sale of user location data [3].

Once a third party collects user data, it is difficult to guarantee that the data are not misused or mishandled. Between 2013 and 2014, Cambridge Analytica collected social media data from Facebook users for academic research, but later repurposed the data for political advertising [4]. In the past decade, major data breaches have exposed billions of user accounts [5]. There are also several instances of malicious apps that directly expose private information without user consent [6]. Regulatory efforts, including the “right to be forgotten” directive under the General Data Protection Regulation, aim to curb this trend in an effort to protect user privacy [7].

These issues present difficulties for biomedical researchers conducting studies that would otherwise benefit from convenient, passive, and longitudinal methods of data collection to identify novel biomarkers and develop digital therapeutics. There is a need for an open and trusted method for sharing data with untrusted third parties that ensures (1) posterior privacy, where personal data are not shared beyond the study for which the individual has consented and (2) that the data are only used for the intended purpose of the study.

In this paper, we reviewed the current state of privacy-preserving techniques for personal data, motivated by a location-sharing use case with applications in health care. We compared privacy-preserving techniques along several axes, including the level of trust required in the research team, the generalizability of the technique, and the availability of open source tool support. It is our intention to provide a pragmatic road map to help researchers make informed decisions about the utilization and processing of sensitive personal data. We provide a reference software implementation for the location-sharing example use case, using one of the examined techniques for privacy preservation.

#### Predictive Modeling in Health Care Using Biomedical and Location Data

Smartphone phone usage, and geolocation data in particular, is consequential for several health care applications. Location data have already been used in a variety of applications in health, for example, to monitor behavioral and environmental risk factors [8,9], to improve disease management and treatment delivery [10], and to inform public health policy in substance abuse [11]. In a representative example, researchers found that features extracted from global positioning system (GPS; movement and locations) and phone usage (social connectedness) strongly related to symptom severity in depression. The availability of smartphone tools provides a vector for continuous, passive assessments that could one day augment current data collection methods in clinical psychopharmacology [12]. However, it is important to stress that although geolocation data can be valuable for health care research, it is also one of the most fundamentally sensitive pieces of personal information.

#### Feature Engineering

Feature engineering is the process of transforming raw data into a representation that is amenable to machine learning algorithms. For example, say you are building a system to forecast driving time between two locations in a major metropolitan area. You are given data that contain the date, time of day, and driving time between the two locations for the previous year. The raw date data (YYYY-MM-DD) are unlikely to be useful for predicting drive time, but knowing whether the day is a weekday or weekend may be very useful. A machine learning scientist might write code that returns true if the date is a weekday and false if it is a weekend. The newly engineered Boolean feature, *weekday*, encodes important domain knowledge—that traffic patterns are different on weekdays compared with weekends—and may improve the accuracy of the predictions from the machine learning model.

Historically, feature engineering has been a manual process, based on the experience and domain expertise of the machine learning scientist [13]. More recently, automated systems that learn feature representations automatically from the data, such as sparse coding and auto encoders, have demonstrated good performance as the basis for deep learning models. Here, we describe a framework for feature engineering that preserves the privacy of identifiable data and is applicable to either manual or automated feature engineering procedures.

#### Minimal Exposure Feature Engineering

**Figure 1:**
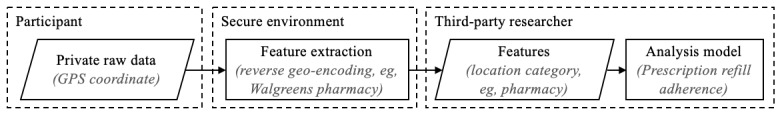
A minimal exposure approach to feature engineering, where sensitive raw data are not exposed to a third party. As an example, reverse geo-encoding is performed in a secure environment to extract a location category, which could be used to determine population models on prescription refill adherence.

Our approach is based on the premise of minimal exposure; that participants should only reveal the minimal data required for the study and researchers should only collect the data required for the study. The feature engineering step of an analysis pipeline offers an opportunity to limit exposure by transforming identifiable, sensitive, or otherwise private data into deidentified or anonymized features. This minimal exposure approach to feature engineering creates a framework that benefits both participants and researchers. By making it openly difficult for researchers to obtain raw personal data, participants may feel more willing to share their data and contribute to research studies. At the same time, removing researcher data access may simplify and expedite research studies by reducing the resources diverted toward maintaining secured data servers and limiting exposure to personally identifying information. In Figure 1, we illustrate the approach whereby raw data and feature extraction are encapsulated in a secure environment, removed from the researchers who are primarily interested in the underlying features.

#### Interest in Blockchain Technology for Data Privacy

Over the course of 6 months in 2018, the landscape map of blockchain projects within the health care sector tripled in size, with nearly 150 projects that raised more than US $660 million [14]. The most common function of health care and biomedical blockchains is the management of data and digital assets (38%), which includes identity management, patient data, health systems operations data, and more [14]. This suggests that one of the more popular applications of blockchain technology centers around the idea that individuals may desire control of their data as a way of feeling that their privacy and data are kept more secure.

A blockchain consists of a distributed network of unaffiliated computers (nodes) that maintain an immutable record of transactions that are verified using a cryptographic protocol. Blockchain networks are further characterized as public, private, or consortium networks depending on who can participate in the network, and how transactions are verified. In public blockchains, transactions are verified and a global state of *truth* (distributed ledger) is maintained by a *trustless* network. A *trustless* network refers to a decentralized network with a consensus protocol. The consensus protocol incorporates sender authenticity via public key cryptography, game theory and cryptoeconomic (digital currency) incentives, and computational complexity to ensure that honest nodes are rewarded, and dishonest nodes are penalized to maintain the canonical truth. By making each transaction auditable and permissionless, public blockchains ensure data integrity, trust, and verifiability.

Advances in blockchain technology have enabled the deployment of rule-based, self-executing software code called smart contracts. Smart contracts remove the need for intermediaries by acting as predefined arbiters. In addition, smart contracts are immutable and publicly verifiable when the contract code is made public. The combination of smart contracts with a *trustless* environment is what eliminates the need for trusted third parties that are responsible for managing private data. These features make smart contracts particularly relevant to this study.

### Aim of This Study

The aim of this study was to examine and compare current privacy-preserving methods based on their ability to maintain the privacy of personal shared data. Methods were compared based on the level of trust required of a third party, and the practicality of implementing these techniques framed as a feature engineering step. This study also aimed to identify the more promising techniques that researchers and software developers can use when building applications concerned with preserving data privacy.

The examination is set against a practical use case of collecting location data from individual participants, from which interesting features related to health can be extracted. To make this example as accessible to researchers as possible, we provide an open-sourced software project that implements one of the examined techniques for the location sharing use case.

## Methods

### Primary Outcomes

The primary outcomes of this study are as follows:

1. Define a set of properties on which to evaluate the privacy-preserving properties of each approach.
2. A qualitative comparison, grounded in a geolocation feature engineering use case, of the privacy-preservation properties of each approach.
3. A proof-of-concept software implementation for extracting the category of a location from GPS coordinate data while maintaining privacy using one of the more practical blockchain techniques.

### Literature Review

We conducted a review of literature, health care–related blockchain use cases, and applied blockchain projects on the Web. These techniques were identified using keyword searches in electronic databases (Google Scholar and PubMed) and search engine (Google) results. The keywords were *privacy blockchain*, *deidentification*, and *privacy feature engineering*. The results at the time of the search (January 2019) consisted of methodologies described in a variety of formats, including 4 academic papers in peer-reviewed journals, 6 academic papers in conference proceedings, 2 literature and product surveys, 1 doctoral dissertation, 7 scientific journal preprints, 11 product specifications, and 1 academic lecture materials.

The techniques were divided into the following categories: (1) methods that rely on a trusted third party, (2) cryptographic methods, (3) trusted execution environments (TEE), and (4) methods incorporating blockchain. Examples about existing implementations of each of these technologies are included in Multimedia Appendix 1 [15-43].

### Evaluation Properties

Data privacy laws [44-46] offer a regulatory perspective on the several dimensions in which data privacy can be compromised. Table 1 summarizes some of the key regulatory principles.

These regulatory guidelines make it clear that data privacy is highly dependent on the responsibilities of trusted organizations, and the capabilities of the technologies they implement. We predict that future data-sharing systems will be informed by these privacy guidelines and that a framework for evaluating privacy-preserving technologies should map to these guidelines. In this paper, each privacy-preserving approach is evaluated based on the following properties:

1. The level of trust required in a third party because of the following:
  - Third-party access to raw data
  - Participant visibility of data use
  - Third-party ability to reuse data
  - Centralization and single points of trust
  - Potential for security vulnerabilities
2. The generalizability and implementation practicality of the technique:
  - Computational or communication complexity
  - Implementation complexity
  - Availability of developer tools
  - Availability of open source tool support

**Table 1:** Data privacy laws in the European Union and the United States.

Source and guideline		Summarized text
**General Data Protection Regulation Article 5**		
	“data minimisation”	Personal data collection is limited to what is necessary
	“lawfulness, fairness and transparency”	Personal data are processed in a transparent manner
	“purpose limitation”	Personal data are collected with an explicit purpose, and further processing adheres to the initial purpose
	“accountability”	Third parties are responsible for adhering to privacy laws
	“integrity and confidentiality”	Personal data are securely processed and there are protections against unauthorized use
**Health Insurance Portability and Accountability Act Privacy Rule**		
	Limits who can view and share an individual’s health information	Health information cannot be used for purposes not directly related to providing health treatment without an individual’s consent (with exceptions)
**Health Information Technology for Economic and Clinical Health Act Subtitle D**		
	Data security of digital health information	Electronic medical records must be secured, and data breaches must be reported

### Description of Geolocation Use Case

Like most complex data types, GPS data are typically transformed before being used in an analysis through feature engineering. There are 2 broad classes of geolocation features that underlie most of the current health care research applications of geolocation data.

#### Statistical Descriptors

They compute summary statistics from the raw GPS data. For example, total distance traveled in a day, the variance in number of locations visited, and the travel radius.

#### Semantic Descriptors

They combine the GPS data with a third-party geospatial information system to determine location types, such as library, gym, or house of worship or broad location themes (eg, neighborhoods with high rates of crime defined by census data).

A few examples of application use cases that would use geolocation features include replacing active monitoring tasks [8-10,47-49], triggering just-in-time interventions [10,49,50], and accessibility to health services [11,51]. Geospatial applications that incorporate blockchain include the management of IoT devices, crowd-sourced data collection, and emergency response [52].

Reference to *geolocation feature extraction* will be made in the Results and Discussion sections to ground the investigation in a practical use case while evaluating different approaches for preserving privacy.

## Results

### Trusted Third-Party Methods

In a traditional biomedical research setting, the protection of human subjects is managed by an institutional review board (IRB) at the research institution. The role of the IRB is to certify that research subjects are informed of the risks of participating in research, data security guidelines are followed, and risks and safeguards are clearly outlined and mitigated. In this model, the research institution operates as a trusted third party with a responsibility to protect patient data privacy. However, new forms of research enabled by smartphone technology, biosensors, and the routine collection of large datasets are changing the nature of research and straining the traditional process whereby a single institution can act as the trusted third party [53]. In the following sections, we cover 2 conventional approaches to privacy preservation that rely on a trusted third party.

#### Server-Side Deidentification

**Figure 2:**
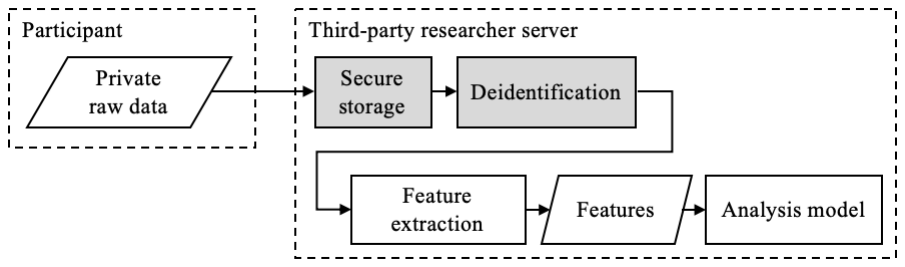
Server-side deidentification: (1) raw data may be exposed during feature extraction and analysis [54], (2) data access control is centrally controlled and mutable, so there is no strict enforcement of how data will be used, and (3) deidentification procedures are often single-use custom software implementations and are unlikely to be open sourced or certified to be thorough and secure. In the case of global positioning system location data, the raw data itself can sometimes be combined with external sources of data (eg, social media) to identify an individual [55-57]. Secure storage and deidentification are highlighted in gray to indicate steps in data pipeline that require trust with handling private data.

A typical server-side data collection pipeline for a research study will ingest raw participant data from a client-side application and incorporate encryption, access control, deidentification procedures, or some other method to ensure that the raw data are not irresponsibly exposed. This approach is straightforward to implement and can afford a research team strict control over the feature engineering pipeline. Software updates can be made server-side without the need to force users to update client-side. However, a high degree of trust must be placed in the research team, as private data are exposed at several stages in the pipeline (Figure 2).

#### Client-Side Feature Extraction

**Figure 3:**
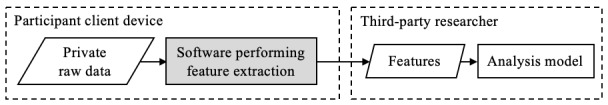
Client-side feature extraction, where the installed software is highlighted in gray to indicate that some level of trust is needed that the software is secure and honest: (1) participants must maintain an updated version of the software so that the feature engineering is appropriate and secure and (2) participants must trust software developers or software validators that the installed application is performing as intended. Open-source software can increase visibility and provide stronger assurances of data privacy but practically requires additional security verification.

Deployment of software that maintains raw data on device and performs feature extraction locally is another viable method for privacy preservation and increasingly a gold standard when it comes to data collected on smartphones. Localizing data on a device so that only the participant has access to it eliminates the risk that third parties can expose, misuse, or repurpose the raw data, but it relies on the integrity of the installed software (Figure 3).

### Cryptographic Techniques

#### Proxy Re-Encryption

**Figure 4:**
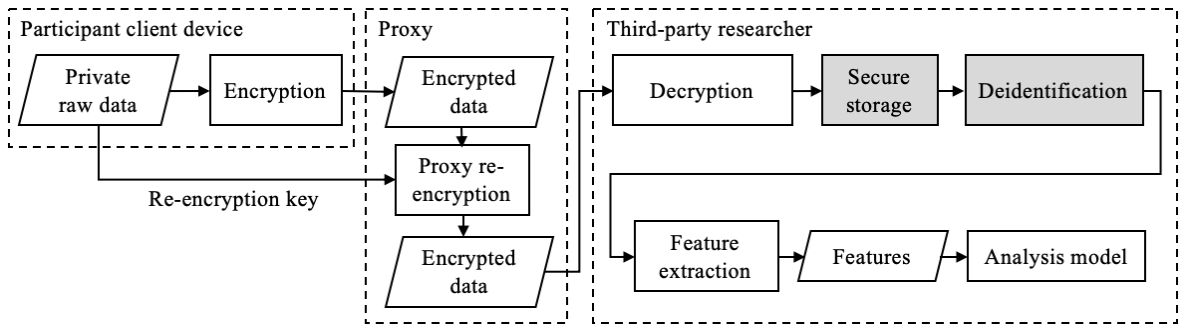
A participant can provide decryption rights to a researcher through proxy re-encryption. The researcher must still be responsible for secure storage and deidentification of the data, along with posterior privacy.

Proxy re-encryption (PRE) is a technique in public-key encryption that allows a proxy to delegate decryption access to encrypted data from one party to another (Figure 4). An important characteristic of PRE is that the proxy does not learn any information about the contents of the encrypted data. As such, it is a powerful data access control technique.

However, PRE is limited in its utility for privacy-preserving feature engineering because data access control does not provide a mechanism for posterior privacy. This still requires trust that the research team will manage the decrypted data securely and honestly.

#### Secure Multiparty Computation

**Figure 5:**
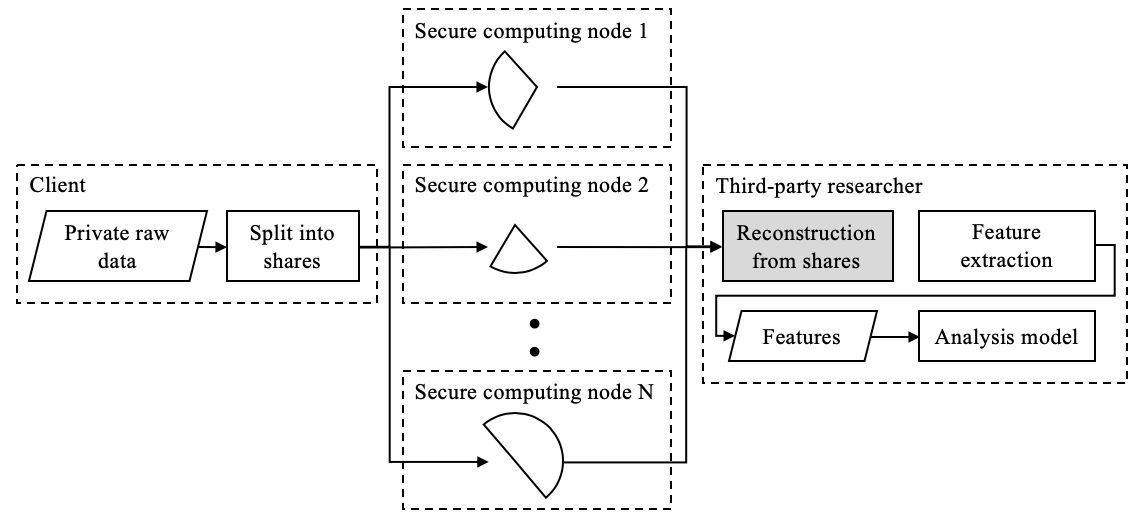
Distributing trust among multiple parties with multiparty computation. In one type of multiparty computation, private data can be decomposed into secret shares and stored on several computing nodes; reconstruction of the private data is only possible with all (or a majority) of the secret shares. As shown in the figure, this alleviates the issue with secure storage of the data but does not secure the data once it is reconstructed. Trust is still required when reconstructing a single participant's private data and is highlighted in gray.

Multiparty computation (MPC) is a class of cryptographic protocols that emulate computations evaluated by a trusted party but instead distributes trust among several parties (Figure 5). MPC is best suited to feature engineering problems that compute a result on aggregate data rather than a single participant’s data. In addition, MPC suffers from being exponential in communication (between parties), which limits performance.

Some implementations tackle private data sharing using MPC protocols and attempt to mitigate security risk further by supporting distributed storage architectures. However, these distributed systems are typically limited in the number of nodes and managed by a single organization (ie, single point of trust). In theory, a single point of trust could have an agenda and exert a degree of control over every node in the distributed network. However, if all the nodes are controlled by a single organization, it is feasible for the organization to access private user data.

The principle behind using a distributed network of computing parties is that there is no single point of trust and is an important one that will be revisited when examining blockchain methods.

#### Homomorphic Encryption

**Figure 6:**
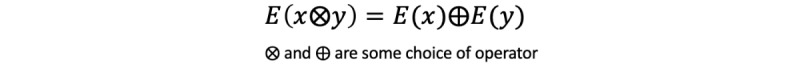
Equation illustrating property of homomorphic encryption, where E(x) represents the encryption of data x.

**Figure 7:**
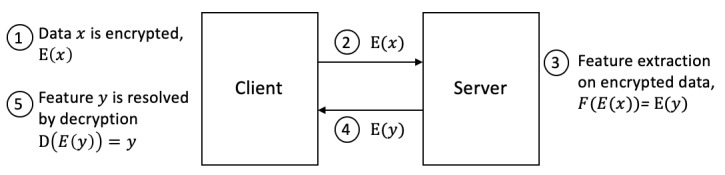
Typical client-server homomorphic encryption (HE) pipeline. Raw data ownership is maintained client-side but inaccessible to other parties. HE is limited for the feature engineering use case because it requires decryption as a last step. If researchers are provided the ability to decrypt the feature data from step 5, then they would also have the ability to decrypt the raw, sensitive data from step 1.

Homomorphic encryption (HE) is a form of encryption where a computation on encrypted data will produce the same result as performing the computation on the unencrypted data before encryption. This can be formalized as illustrated in Figure 6. There are different schemes of HE, including partial and fully HE; partial homomorphic encryption (PHE) indicates one or more operations can be run on encrypted data and preserve homomorphic properties, while any arbitrary computation is possible when dealing with fully homomorphic encryption (FHE). HE is strong from a privacy-preserving standpoint because a participant can maintain exclusive ownership rights over data but potentially limits the feasibility of feature extraction (Figure 7).

In a data-sharing context, HE may be suitable in specific use cases for feature extraction when the encrypted data vector is an interesting feature itself. There is also a broader applicability for privacy-preserving feature extraction when features from individual participants are not required, and data can be aggregated in the encrypted domain. However, the applicability of HE is not suitable for general purpose feature engineering in its current state and depends on the nature of the data and of the feature extraction. For example, resolving a location type from a GPS coordinate is not computable but rather the result of a lookup function. HE would not serve this kind of scheme.

Another major hurdle in the adoption of HE is the increased computational complexity of processing encrypted data, resulting in extremely long processing times. Sophisticated computations cannot be achieved with PHE, and FHE suffers from very low computational performance [17]. This makes all but the simplest operations impractical using HE.

#### Zero-Knowledge Proof

A zero-knowledge proof (ZKP) is a cryptographic method whereby a *Prover* convinces a *Verifier* whether some mathematical statement is true or false, without revealing any underlying data. Privacy preservation is built into ZKPs, making it a strong approach for handling private data.

ZKPs can be further classified based on 2 kinds of statements that should be proved in *zero knowledge*: statements about facts (eg, that a participant’s GPS coordinate corresponds to a *hospital*) and statements about knowledge (eg, that a participant’s GPS coordinate is known) [19]. The latter kind is a ZKP of knowledge and is the most common application of ZKPs, which is identification and authentication (eg, password authentication). However, in the context of feature engineering, it is the former type of problem that extracts some metadata that is relevant.

**Figure 8:**
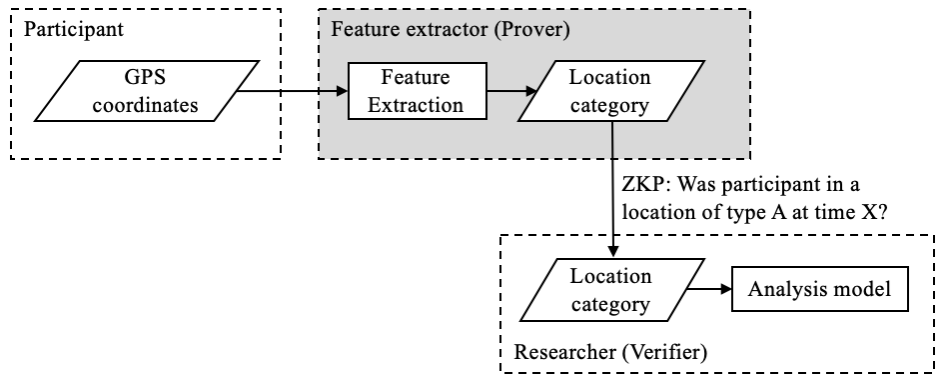
Hiding global positioning system (GPS) coordinates behind a simplistic zero-knowledge proof (ZKP) implementation. Practically, this looks like a simplistic black box whereby the ZKP is a subroutine that performs feature extraction without revealing the raw GPS coordinate to the researcher. For example, this subroutine could consist of implementing a lookup table that maps GPS coordinates to category of location. The challenge then lies in careful implementation and permeability of this subroutine to ensure security of the data (highlighted in gray). A malicious party should not be able to identify a participant’s GPS coordinates through a process of elimination by trying several inputs. The analogue would be trying to guess a password via brute force.

The ZKP concept closely mirrors the minimal exposure framework described in Figure 1. To address the geolocation feature extraction use case, a data pipeline as shown in Figure 8 could be implemented.

As ZKPs more broadly correspond to a variety of techniques, it is difficult to recommend it summarily for general feature extraction problems, and they should instead be evaluated on a case-by-case basis. In addition, some common challenges include implementation and computational complexity. Sometimes, they still require a trusted third party to prove a statement [58]. It is our view that ZKPs hold potential as building blocks for privacy-preserving protocols but is currently an active area of research [59] rather than a practical and accessible tool for implementation.

### Trusted Execution Environments

**Figure 9:**
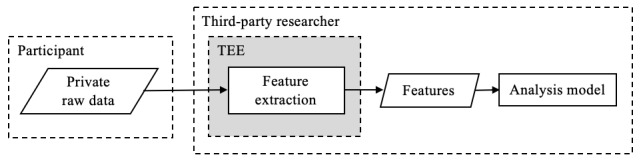
A trusted execution environment (TEE; highlighted in gray) provides data privacy through encapsulation. Security features include memory isolation, memory encryption, isolated architecture, and secure key provisioning. A remote attestation process follows to verify correct execution of a program and provide a proof of origin [60]. A level of trust is still required in TEEs (highlighted in gray) because of the risk of hardware exploits.

A TEE (also referred to as secure hardware enclave) is a chip-level hardware design in modern processors that enables isolated execution over confidential data. Figure 9 illustrates how TEEs could encapsulate private raw data without exposing to the researcher. A major benefit of TEEs is that they have little performance overhead over native computations, making them practical for a wide range of applications [25], while providing guarantees that malicious applications cannot tamper with the computations running on secure enclaves.

An important consideration when using TEEs is the very real risk of hardware vulnerabilities that can be exploited. In early 2018, hardware vulnerabilities in modern commercial processors were reported that can expose private data to rogue processes (Meltdown) or attacks on processors that perform branch prediction (Spectre) [61]. Another vulnerability (Foreshadow) explicitly affected Intel SGX processors [62], bringing to question how trustworthy a secure enclave can be. In an effort to address these concerns, emerging open-source TEE projects argue that security by obscurity in commercial designs is insufficient and that community-driven, open security will lead to more reliable designs [25].

Another criticism of TEEs regarding data privacy is that practical applications might use a single or handful of TEEs, which centralizes the management of data. The thought is that it is still a “very strong assumption to require all participants to globally trust a single or handful of (TEE) processors” [63]. To address this limitation, some projects have emerged that incorporate blockchain with TEEs to decentralize the network of computing nodes. This approach will be examined further in the next section on blockchain methods.

### Blockchain Methods

#### Private and Consortium Blockchains

**Figure 10:**
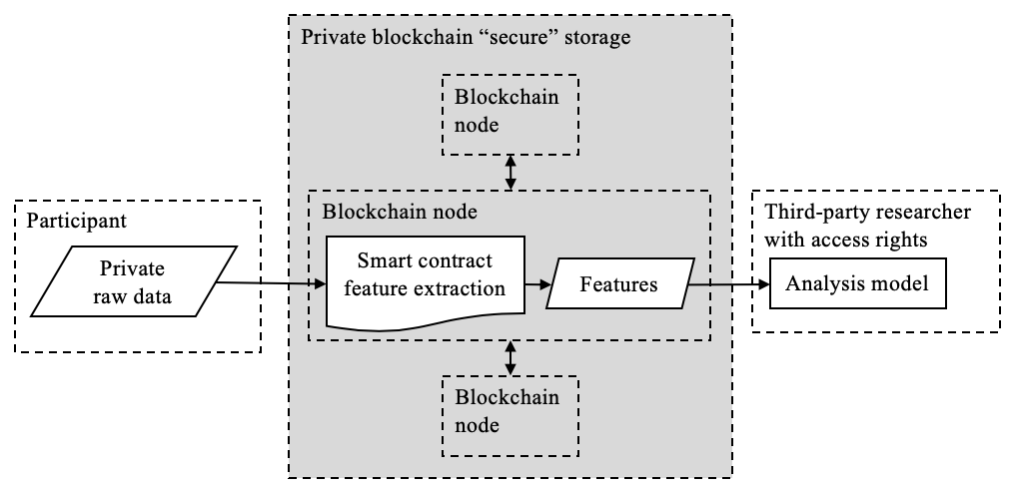
Private blockchain where it represents a secure data environment but requires similar trust as other centralized techniques (participant consent to release data, trusted parties). For this reason, the entire private blockchain network is highlighted in gray.

Private and consortium blockchain networks limit who can participate in the network, usually by creating access controls that are managed by highly trusted entities. Often, additional rules are included to create a permissions system, control which nodes can verify transactions, and make transaction data private to the parties involved. The last of the reasons is particularly appealing in a privacy-centric data–sharing context, but it comes at the price of placing trust in the maintainers of the private network. However, the very same feature that makes public blockchains so appealing—a trustless, decentralized network state secured by cryptoeconomic incentives—is missing from private blockchains and is illustrated in Figure 10.

#### Public Blockchain Smart Contract

Smart contracts on a public blockchain are small, modular pieces of software that cannot be changed once they are deployed on the network. This is an advantageous quality for privacy-preserving software, because a user of the smart contract is guaranteed that their data will always be processed the same way. The functionality of deployed smart contracts is verifiable when the smart contract code is made public.

**Figure 11:**
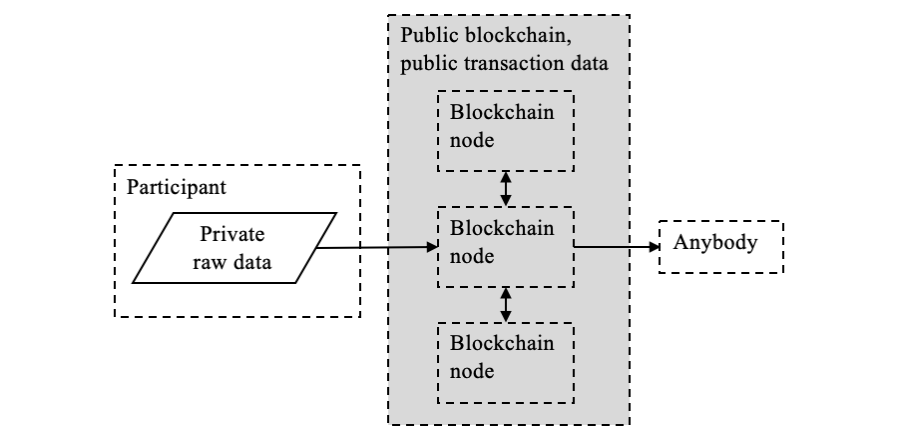
Public blockchain implementation, where transaction data are public. The entire network is highlighted in gray to indicate data exposure. The standard workaround is to ensure that any sensitive data recorded are encrypted. As a result, it is generally impractical to run feature extraction on a smart contract and would require some off-chain computation on a centralized server that requires trust.

However, the first important acknowledgment when considering traditional public blockchains (eg, Ethereum) for data sharing should be that input data uploaded to a public blockchain is publicly visible and permanently recorded. This allows for all fund senders and recipients, all transaction data, and the state of every contract variable to be visible to any observer, as illustrated in Figure 11.

One argument is that blockchains offer privacy because the originator and recipient of data transactions are described only by a randomly generated account address. Therefore, pseudonymity is possible if participants generate new addresses for each transaction. However, Web trackers have shown it is possible to deanonymize users by analyzing transactions [64], and in the case of certain sensitive data such as GPS coordinates, it is possible to reidentify a large fraction of users by comparison with other structured data available (eg, from social media) [33,55-57].

#### Privacy-Preserving Blockchains Incorporating Zero-Knowledge Proofs

The allure behind incorporating ZKPs with a public blockchain is to enable data privacy while maintaining the benefits of blockchain: no single point of trust and immutability of transactions. One implementation of this technique that captured public attention was the ability to hide the origin, destination, and amount in a cryptocurrency transaction [18,34,35].

The idea of extending ZKPs on blockchain to include smart contract logic would be a powerful catalyst for privacy-preserving, trustless applications. A blockchain proposal called Hawk [33] uses ZKPs to verify transactions and executes private smart contracts off-chain. Unfortunately, Hawk cannot guarantee posterior privacy because it relies on a minimally trusted manager, which is disincentivized from revealing sensitive data during transactions but provides no guarantees after the transactions are complete. In addition, the Hawk paper has yet to materialize into a usable software release. Similarly, there are also no details that describe if and when private smart contracts on Ethereum will be available in the near future.

The limitation of ZKP computational complexity is heightened in the context of blockchain, which requires the technology to be deployed at the distributed scale. ZKPs on blockchain is an active research area, making private data sharing and feature engineering in this context inaccessible for the time being. An application for sharing GPS coordinate data using ZKPs with blockchain smart contracts will have to wait for the technology to develop.

#### Privacy-Preserving Blockchains Incorporating Trusted Execution Environments

**Figure 12:**
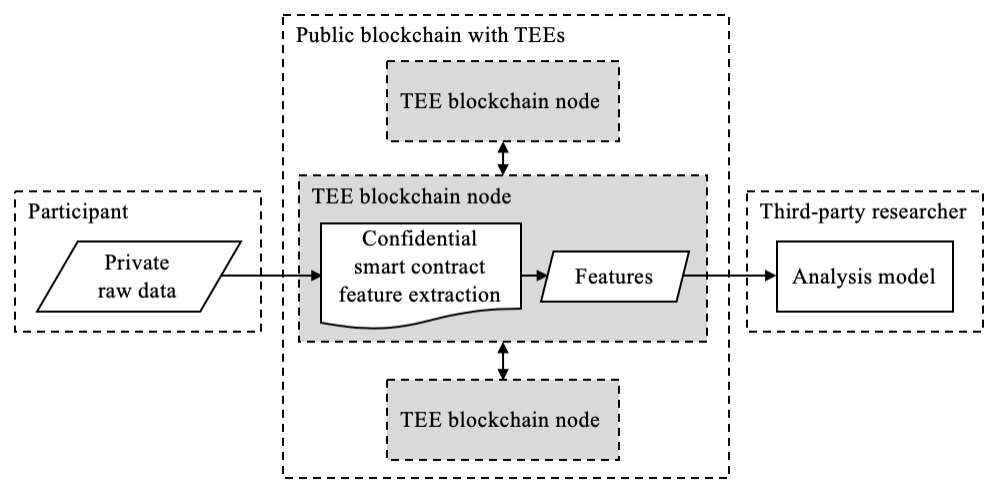
Blockchain that incorporates confidential smart contracts on trusted execution environments (TEEs) for feature extraction. Similar to single TEEs, the risk of hardware exploits should not be ignored.

In an effort to encourage less data siloing, platforms are emerging that hybridize TEEs and blockchain smart contracts. This approach boasts the strengths of modular, immutable software with isolated computation environments so that the data pipeline is transparent and secure. This technique is illustrated in Figure 12.

Nevertheless, this approach still hinges on the security of the underlying TEE hardware and its vulnerabilities. Platforms that incorporate blockchain and TEEs are new technologies, and experimental by nature, so underlying security threats remain to be uncovered.

This approach also provides benefits in terms of implementation practicality and accessibility. The Oasis [32] and Enigma [36] projects are developer friendly, releasing documentation, tutorials, and testnets on which to develop and deploy applications.

### Software Implementation of Location-Sharing Use Case

In this paper, generalizability and practicality of implementation were two of the evaluation criteria used when comparing privacy-preserving techniques. To further evaluate our findings that the hybrid blockchain-TEE technique provides a practical platform for developing privacy-preserving software, we implemented a proof-of-concept software application that addresses the location-sharing use case that frames this paper. This use case is based on the scenario where a research study participant shares useful features about their location data with a third-party research team, without revealing their raw GPS coordinates.

The implementation consists of the following:

1. A smart contract deployed on the Oasis Devnet.
2. A smartphone (iOS) app with a graphical user interface for participants and third parties to interact with the smart contract.

Confidential smart contracts on the Oasis Devnet enable private transaction data and private smart contract state (Table 2), which are used to maintain participant confidentiality and can be used to conceal a participant’s raw geolocation data. The Oasis Devnet manages per-session encryption keys that are used to encrypt communication between a client and smart contract instance, such that nobody else can view the unencrypted transaction data [65].

**Figure 13:**
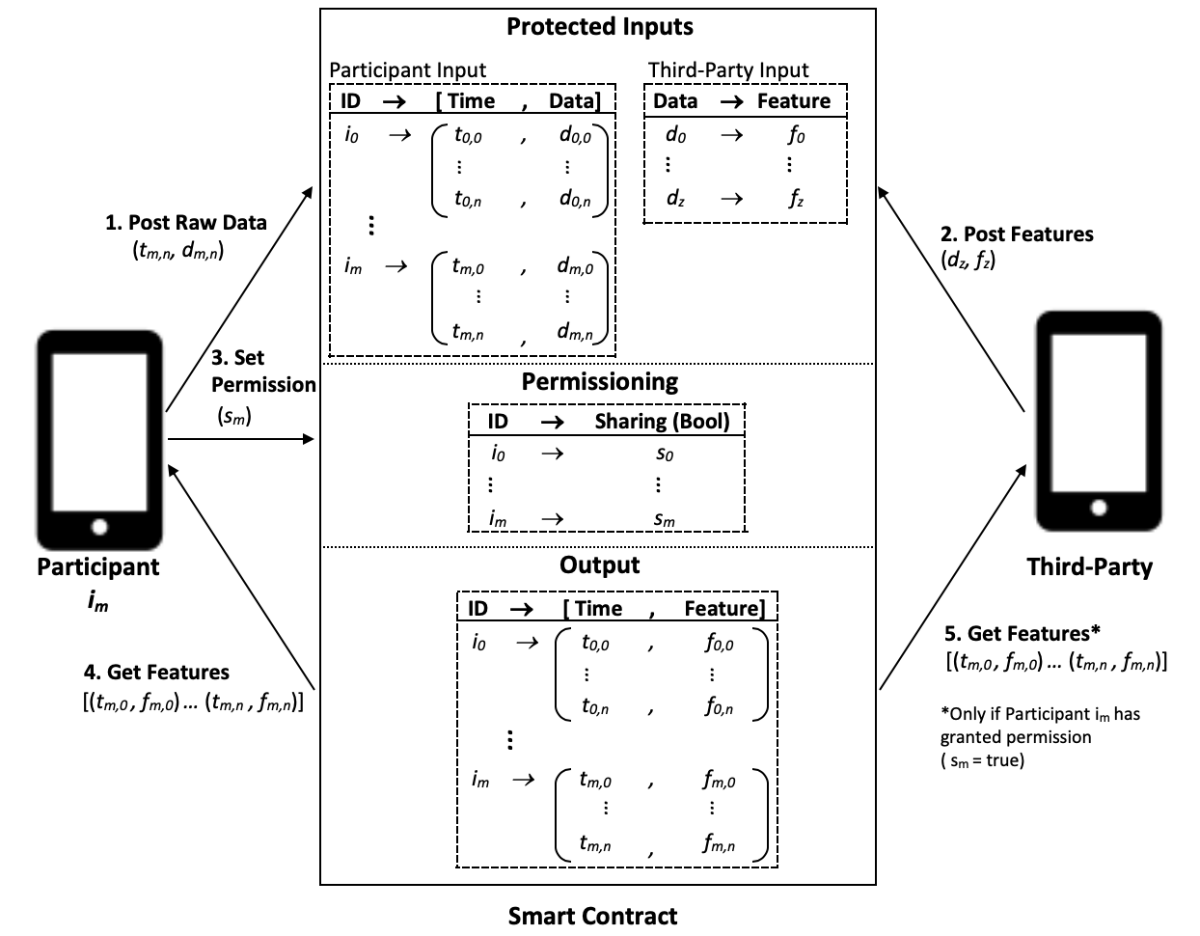
Implementation of the proof-of-concept software application that would address a location sharing use case. A participant, identified by an identifier im, would (1) post their raw identifying location data, dm,n, along with its respective timestamp, tm,n using their iOS device. A third party, from another iOS device, is able to (2) post a raw location data, dz, with a respective feature, fz. For example, a feature such as the category of a location like the string Hospital or Pharmacy. If the location data, dm,n, posted by the participant matches the location data, dz, posted by the third party then the participant’s respective timestamp, tm,n, is mapped to that respective feature, fz. The participant is, at any time, able to (3) set the sharing permission, sm, of all of their previously posted timestamps, tm,0…tm,n, and associated features, fm,0…fm,n, to third parties. The participant is also able to (4) get all of their previously posted timestamps, tm,0…tm,n, and associated features, fm,0…fm,n. The third party is also able to (5) get these same timestamps, tm,0…tm,n, and associated features, fm,0…fm,n if and only if the participant has granted permission, sm=true.

Figure 13 illustrates user interactions with the deployed smart contract. The contract provides a publicly accessible method for participants to post their timestamped location data. Participant identity is kept confidential by maintaining a private mapping in the smart contract state between participant wallet address and a participant identifier.

The smart contract also provides a publicly accessible method for third parties to register a geocoordinate with a predetermined category of location (ie, *hospital*, *gym*, or *pharmacy*). Third parties can query the smart contract to view participant visits to categories of location. This data could be used to build a model of participant visits to registered pharmacies, for example.

**Table 2:** Information visibility on Oasis Devnet.

Visibility	Information
Public	Transaction sender address (ie, participant wallet address and third-party wallet address) Transaction recipient address (ie, smart contract address) Transaction value transaction (ie, amount in *DEV*, the Oasis Devnet token used to fund transactions)
Private	Transaction argument data (ie, raw GPS^a^ coordinate data) Transaction result data (ie, returned feature data) Method name called by a transaction (ie, “postParticipantLocation”) Smart contract state (ie, mapping of participant wallet addresses to participant ID) Event data (not used for this prototype; events (logs) can be emitted in response to transactions)

^a^GPS: global positioning system.

The smart contract is currently deployed on the Oasis Devnet as a traditional smart contract, not as a *confidential* smart contract. Only *confidential* smart contracts run on TEEs and maintain the privacy of smart contract state values and transactional data. However, the library (web3swift) for deploying smart contracts from an iOS smartphone app does not yet support *confidential* smart contract deployment to the Oasis Devnet at the time of this writing. We plan to implement support for this in the near future when web3swift library support is available.

In addition, we hope this proof-of-concept software can serve as a starting ground for future research interested in privacy preservation for feature engineering use cases. Additional details about the software design, development stack, implementation, and trade-offs are described in a tutorial manuscript currently under review [66]. The software source code is publicly available on GitHub at HD2i/GeolocationSmartContract [67] and HD2i/Geolocation-iOS [68]. Full details on the usage are included with the software.

## Discussion

### Comparison of Privacy-Preserving Methods

We have found that conventional methods that rely on a trusted third party for securing participant data generally fall short of providing full guarantees that sensitive data cannot be accessed for unintended purposes. Participants must provide a high degree of trust in researchers that use server-side deidentification procedures and maintain data warehouses themselves. Numerous data breaches on centralized servers in the past decade have illustrated that the responsible question to ask oneself is when, rather than if, private information will be exposed. To combat this, researchers should try to limit the exposure of raw data. A valid approach is to instead perform client-side feature extraction on a personal device under the control of each participant, such as a personal smartphone or private data server. The main drawback to this approach is a high burden for researchers to develop secure, validated software; meanwhile, participants still need to trust that the software is only collecting the intended data and that there are no other data collection routines present that are only described in the fine print of a privacy policy.

Several sophisticated classes of cryptographic techniques exist that provide privacy-preserving methods, and this is an active area of research. PRE can be used for access control to encrypted data, but it falls short of guaranteeing posterior privacy. Secure MPC removes the need for a trusted third party when collecting encrypted data from 2 or more parties and computing aggregate results. However, practical implementations concerned with performance rely on a small number of computing nodes that are managed by a single party, which requires trust in the operators, software, and the security of the computing nodes. HE is considered a holy grail of privacy-preserving methods, under which computations can be performed on private data in the encrypted space. However, there are few use cases where encrypted data can be used as a feature, or applications are limited by computational performance. ZKPs are a broad class of cryptographic techniques that provide powerful guarantees of data privacy but need to be evaluated for a particular application. At a superficial level, they can be used for authentication, but other applications usually incur implementation and computational complexity.

Advances in computing hardware have enabled privacy-preservation through the design of TEEs. These chip-level designs create an isolated memory space where computations can be performed on sensitive data. However, as with software data breaches, it is difficult to guarantee that no hardware vulnerabilities can be exploited. In addition, a participant must still trust that the software running on the TEE computing node is the one advertised.

The previous 3 approaches all share a common element: the feature extraction runs on a centralized server or computing node that is managed by a trusted third party. Although some methods provide higher levels of data security, there is still a shortcoming in terms of visibility. Participants must trust that a third party is doing what it says it is doing, and nothing else. This is where blockchain technology provides a unique benefit.

Blockchains provide a trustless environment that features visibility and immutability by running on a decentralized network that is secure from tampering through cryptoeconomic incentives. In addition, they offer the unique benefit of being immutable pieces of software (smart contracts) that can be verified to do exactly what is promised if the contract code is made public. Of course, this is only guaranteed in public blockchains, where private or consortium blockchains tend to incorporate trusted parties and are more centralized. Unfortunately, blockchains were designed for security and data integrity but not for data privacy.

The combination of cryptographic techniques and TEEs with blockchain addresses the single point of trust weakness and holds the highest potential for trustworthy privacy-preserving platforms. Two promising hybrids are blockchains incorporating ZKPs and blockchains incorporating TEEs. Blockchain with ZKPs has been successful in providing transactional privacy with cryptocurrency, but it has not developed to the same level for smart contract data privacy. Blockchain with TEEs is a developing technology, but it has reached a level of maturity where developers can begin to develop and deploy real applications on these platforms. Naturally, potential hardware vulnerabilities on these platforms do not make this approach ideal; however, until cryptographic methods like FHE can be widely applied at scale, blockchains with TEEs seem to be the best approach available currently. In addition, we found that robust developer documentation and tools were available, which makes this approach accessible for product implementations as well. Our software implementation for the geolocation use case was in part encouraged by the practical direction and developer support provided by the Oasis platform and illustrates that privacy-preserving methods are realizable today on nonproduction developer networks. However, it is important to stress that blockchains with TEE developer networks are experimental at the time of this submission, and a conservative approach should be taken.

Table 3 summarizes our findings and attempts to qualitatively compare each approach in terms of how much trust must be placed in other entities. In addition, a rough indication of each method’s practicality (based on implementation and computational complexity) is defined. Examples of real-world implementations or developing projects for each method are also identified; more detail is provided in Multimedia Appendix 1. Finally, the major limitations of each method are summarized.

**Table 3:** Comparison of trusted third party, cryptographic, and blockchain approaches to data privacy for research studies.

Method	Level of trust	Practicality	Limitations	Examples
Server-side deidentification	High	Medium	Centralized; vulnerable to data reuse and data breaches; no visibility	Strava GPS^a^ devices
Client-side feature extraction	Medium	Medium	No visibility	Apple device predictive keyboard; Open PDS/SafeAnswers
Proxy re-encryption	High	Low	Only for data access control; vulnerable to data reuse and data breaches	NuCypher pyUmbral
Multiparty computation	Low	Medium	Specific use cases; centralized; no visibility; communication complexity	Jana, Sharemind, Partisia, Sepior
Homomorphic encryption	Low	Low	Limited operations or extremely low performance	NuCypher nuFHE
Zero-knowledge proof (ZKP)	Low	Low	Specific use cases; centralized; no visibility; implementation and computational complexity	zk-SNARK
Trusted execution environment (TEE)	Low	Medium	Potential for hardware vulnerabilities; no visibility	Intel SGX, ARM TrustZone, Keystone Project
Private or consortium blockchain	Medium	Medium	Pseudocentralized; depends on design	Hyperledger Fabric
Public blockchain smart contract	High	Medium	Only for data access control	Ethereum
Public blockchain with ZKPs	High	Low	Proof of concept, no software release available	ZCash, Hawk
Public blockchain with TEE	Lowest	Medium	Potential for hardware or other vulnerabilities; nonproduction stages	Enigma, Oasis

^a^GPS: global positioning system.

### Limitations

In the introductory section on minimal exposure feature engineering, we identified that the feature engineering step in an analysis pipeline offers an opportunity to limit exposure and remove identifiable features. Although we promote this framework for minimizing the exposure of private data where possible, we recognize that not all feature engineering problems are amenable to deidentification. In these scenarios, we recommend that data security safeguards be in place, including encryption and secured servers.

Transactions on a public blockchain network have an inherent cost, which the parties involved in the transaction must pay for in cryptocurrency. How large this financial cost is varies based on the value of the cryptocurrency and on the congestion of the network at any given time, so no quantifiable amount is provided here. The cost may be a significant consideration for practical implementations, but our focus was on identifying methods that maintain privacy.

When posting transactions to any internet-connected network, including public blockchains, a reasonable concern is that a participant would reveal their internet protocol (IP) address, which itself is a piece of identifying information. One workaround to this concern would be to implement an internet request proxy (eg, a thin-client of the Tor software), which can relay internet traffic to conceal a user’s location and usage [69]. However, the authors have not implemented this feature, and it is left to future work.

### Conclusions

We believe that the boundaries of data privacy are being pushed forward with blockchain technology. The fundamental limitation of privacy-preserving protocols that run on a single point of trust with centralized servers is addressed by immutable smart contracts. As different cryptographic and software techniques overlap with blockchain, stronger guarantees of privacy become possible. In particular, we think the combination of blockchain with TEEs seems like a practical and forward-thinking approach to privacy-preserving feature engineering. This conclusion is supported by our development and deployment of a proof-of-concept private geolocation data-sharing software on a hybrid blockchain-TEE developer platform. However, no system is free from all vulnerabilities and should be thoroughly tested when interacting with highly sensitive, private data such as GPS coordinates or other biomedical data.

## Appendix

Multimedia Appendix 1 Examples of existing privacy-preserving technologies.

## Abbreviations

FHE: fully homomorphic encryption
GPS: global positioning system
HE: homomorphic encryption
IoT: internet of things
MPC: multiparty computation
PHE: partial homomorphic encryption
PRE: proxy re-encryption
TEE: trusted execution environment
ZKP: zero-knowledge proof
