# Supplementary material for: Privacy-Preserving Methods for Feature Engineering Using Blockchain: Review, Evaluation, and Proof of Concept
Source: J Med Internet Res. 2019 Aug 14;21(8):e13600. doi: 10.2196/13600 (PMC6712958; doi:10.2196/13600)
Supplement: Multimedia Appendix 1 [file jmir_v21i8e13600_app1.pdf]

Multimedia Appendix 1: Examples of existing privacy-preserving technologies

The following sections identify a number of examples of existing technologies from each of the techniques described in the results section of this paper.

Trusted Third-Party Methods

Server-Side Deidentification

Data from wearable global positioning system (GPS) trackers is one example where raw data is stored server-side. For example, GPS devices record GPS coordinate time series data [38], and data servers compute statistical features like distance traveled. Users of a GPS tracker may authorize third parties to download deidentified coordinate data from the data servers, but are still subject to the data server security, access control and de-identification protocols.

Client-side feature extraction

Modern smartphones such as the Apple iPhone perform on-device feature extraction of text data to enable predictive keyboards [39]. In this particular example, even the feature data never leaves the device, but serves to illustrate a practical application where raw user data never needs to be stored on a third party's centralized server.

Cryptographic techniques

Proxy Re-encryption (PRE)

The primary application for PRE has been for secured distributed data storage, and research in the field has focused on improving security and performance [22], revocable access control through key rotation [23], and the ability to re-encrypt data multiple times using fully homomorphic encryption [15]. Recent implementations have included bolstering the technique with distributed consensus networks (i.e. blockchain) to decrease the trust required of any single proxy [24].

Secure Multiparty Computation (MPC)

A few practical applications and pilot projects that incorporate MPC include [21]:

1. Secured databases which can incorporate query re-writing over encrypted data stored on relational databases, or additive secret sharing over distributed databases for data analytics
2. Access key management
3. Statistical computations over private data

The security of most MPC protocols is characterized by a security model which can tolerate a certain number of dishonest “adversaries”. For example, SPDZ is a popular MPC protocol that can tolerate up to all but one party being dishonest [40].

Homomorphic Encryption (HE)

There are a handful of examples where HE has been applied to specific use cases for feature extraction when the encrypted data vector is an interesting feature itself. For example, deep neural networks have been applied directly to encrypted data in use cases such as biometric authentication [16] and optical character recognition [17].

FHE has also been used in conjunction with MPC to hide individual input data [40], while computing an aggregate result in the encrypted space. A shared decryption step reveals the result.

Since computational complexity is one of the main drawbacks for FHE, speeding up computations is an active research area, and some promising improvements have been achieved using GPUs to parallelize computations [20].

Zero-Knowledge Proof (ZKP)

Zero-knowledge proofs of knowledge are particularly applicable in problems dealing with identity and authentication. Blum, Feldman and Micali developed a scheme where zero-knowledge was obtainable without interaction by sharing a common reference string between a Prover and a Verifier [43]. Another development coined zk-SNARKS has been incorporated into the verification of blockchain transactions, explained in the corresponding section.

In general, problems that require a statement of fact (e.g. some number N exists in the set of composite numbers) could addressed using a ZKP technique through the following steps: (i) representing the problem as a Boolean circuit, where the circuit is only satisfied if and only if you know the correct input, (ii) translating the circuit into graph problem, and (iii) solve the graph problem using the Goldreich, Micali and Wigderson (GMW) protocol [19, 37]. However, these steps are technically so involved and complex as to make this infeasible for most applications.

Trusted Execution Environments (TEE)

Several implementations of TEE exist or are in development, including commercial technologies like Intel SGX [26] and ARM TrustZone [27] processors, and open-source technologies on RISC-V based processors like the Keystone project [25].

A few pilot projects incorporating TEEs include securing cryptocurrency wallets [29], running queries on genetic data [28], and blockchain based cloud computing platforms [30-32]. Since TEEs support running compiled software programs, it would also be possible to develop a feature extraction routine for GPS data that could be run on a TEE.

Blockchain Methods

Private and Consortium blockchains

Several implementations that incorporate private blockchain address health data sharing needs such as data access to research study data [41] or data integrity and tamper-resistance [42]. The two most popular private blockchain platforms are forked Ethereum blockchains (adapted to run separately from the public, main Ethereum network), and Hyperledger.

Privacy-preserving blockchains incorporating Zero Knowledge Proofs

Before 2016, most blockchain innovations concerned with privacy focused on cryptocurrency transactions. ZCash [18] is a blockchain implementation that uses ZKPs to hide a payment's origin, destination, and transaction amount, while maintaining the ability to verify transactions. Ethereum later incorporated cryptographic primitives that support ZCash-style ZKPs on the Ethereum Byzantium version release, and private cryptocurrency transactions were verified on test networks in late 2017 [34, 35].

Similar to the delayed status of the Hawk blockchain [33], there do not seem to be implementable solutions which incorporate privacy-preserving smart contract logic based solely on ZKPs.

Privacy-preserving blockchains incorporating TEEs

The Enigma platform [30, 36] combines private off-chain data stored on a peer-to-peer distributed hash table, Multi-Party Computation on Intel SGX processor, and a public blockchain that holds reference to the data. The Ekiden / Oasis protocol [31, 32] also uses a hybrid blockchain and TEE model, and aims to support multiple TEEs including Intel SGX and the open-sourced Keystone project [25]. Both projects suggest that research is underway to incorporate cryptographic techniques like ZKPs and MPC in their protocols.
